# Peer Relationship Experiences Of Deaf And Hard-Of-Hearing Adolescents

**DOI:** 10.1093/deafed/enz048

**Published:** 2020-02-12

**Authors:** E Terlektsi, J Kreppner, M Mahon, S Worsfold, Colin R Kennedy

**Affiliations:** 1 Faculty of Medicine, University of Southampton; 2 School of Education, University of Birmingham; 3 School of Psychology, University of Southampton; 4 Division of Psychology and Language Sciences, UCL

## Abstract

Deaf and hard-of-hearing adolescents (DHH) experience more peer problems and lower levels of friendships than their hearing peers. This study used a qualitative approach to identify their experiences of peer problems and factors influencing them. A sample of 30, 13–19 year-old DHH adolescents with a moderate to profound hearing loss, drawn from a population-based cohort study in which their receptive language and social–emotional skills had been assessed, underwent semi-structured interviews. Interviews were analyzed using thematic analysis. Participants reported that, overall, they had developed positive and rewarding relationships with their peers, notwithstanding their earlier experience of being bullied. Conflicts and infrequency of interaction in their friendships were mainly reported by girls. Adolescents with moderate hearing loss were identified as facing the same or even more barriers than adolescents with severe to profound hearing loss in making new friends. Implications for educational practice are discussed.

Peer relationships are important predictors of academic and life skills in children and young people (Von Hohendorff, Couto, & Prati, 2013; Webster-Stratton & Reid, 2004). Peer relationships are particularly salient during adolescence when young people spend much of their time with peers, often turning to peers rather than parents for intimate disclosures, and seeking feedback from peers as an important input to the development of their sense of self (Prinstein & Giletta, 2016). This developmental period may be especially challenging for those who are deaf and hard of hearing (DHH) (Rich et al., 2013) as they face significant changes associated with puberty and adolescence in a hearing world where communication and access to information and peers can be compromised by their hearing loss (Brice & Strauss, 2016).

The current study examines how a heterogeneous group of DHH adolescents experience peer relationships and friendships. A distinction is made here between peer relationships and friendship, with the latter being a subtype of peer relationships. Peer acceptance and popularity define peer relationships, whereas friendship is defined as a close, mutual, dyadic relationship (Hartrup, 1996). The peer relationships and friendships of deaf adolescents might be influenced by both individual’s characteristics (e.g., gender, degree of hearing loss, type of amplification, language, and communication) and contextual factors (e.g., education placement, family characteristics).

## Peer Relationships of DHH Adolescents with Hearing Peers

In the context of the global increase in inclusive education, recent research has focused on the interactions of children who are DHH with their hearing peers. One longitudinal study reported that, in spite of being no different from hearing adolescents with regard to social skills and behavior, DHH adolescents felt less secure than their hearing peers and faced more difficulties in making friends (Antia et al., 2011). Similarly, a qualitative analysis of interviews with parents, teachers and DHH adolescents themselves found that adolescents were worried about peer relationships and struggled with their concept of self (Punch & Hyde, 2011).

A recent systematic review and meta-analysis of 45 studies identified higher rates of emotional and behavioral difficulties in DHH children and young people compared to hearing peers (Stevenson et al., 2015). Across the studies identified by this review, the scores for parent, teacher, and self-rating of peer problems consistently indicated more peer problems in DHH children than in their hearing peers. Subsequent to that systematic review, a further study using the Strengths and Difficulties Questionnaire (SDQ) (Goodman & Scott, 1999) confirmed that DHH adolescents with no long-term health conditions other than their hearing loss identified themselves as experiencing a significantly higher level of problems with peers and friends compared to a hearing comparison group, whereas the two groups had similar group mean scores on other SDQ subscales (Stevenson et al., 2017). This suggests that DHH adolescents perceive peer problems as the most salient issue regarding their emotional and behavioral health. This conclusion was also supported by a systematic review of 21 papers on the interactions between DHH children and young people with their hearing peers across primary and secondary education, which reported that DHH children experience difficulties with communication and with initiating, entering, and maintaining interactions with their hearing peers (Xie et al., 2014).

## Quality of Friendship

A subtype of peer relationships is friendship. Friendships are dyadic and reciprocated relationships with peers characterized by shared enjoyment, mutual liking, commitment to each other and closeness (Bukowski et al., 1996). Friendship can have both positive (e.g., loyalty, intimacy, prosocial behavior, self-esteem support) and negative (e.g., conflict, rivalry) features (Berndt, 2002). Studies with hearing children demonstrate that friendships characterized by high levels of positive features are a significant predictor of good social-emotional skills (Bagwell & Schmidt, 2011; Schwartz et al. 2000). Moreover, such high-quality friendships have been found to be protective against being bullied by peers (Bollmer et al. 2005).

Studies on the quality of friendship among DHH adolescents are scarce and report inconsistent results. A small study investigating the effect of quality of friendship on the well-being of 12 Dutch DHH 13–19-year olds, found that, compared to their hearing peers, DHH adolescents scored lower on positive qualities of friendships, such as intimacy and companionship (Wolters & Isarin, 2015). Similarly, a longitudinal study of Dutch DHH children and adolescents aged 9–16 years, of whom 77% used spoken language, reported that their friendships were characterized by more negative features (e.g., conflicts) and fewer positive features (e.g., companionship) than those of hearing children (Kouwenberg, 2013). In addition, a recent Dutch study of the effect of emotional awareness of DHH 9–15-year olds in either special or mainstream education on the quality of their friendships found that DHH adolescents in special education reported lower levels of positive friendship features on rating scales compared to DHH adolescents in mainstream education. However, the latter group’s levels of positive friendship features were also lower than those of their hearing peers. In addition, the study highlighted that emotional awareness and control were important correlates of positive friendship features, areas in which DHH children showed deficits compared to hearing peers (Rieffe et al., 2018). All the above three Dutch studies demonstrated group differences between hearing and DHH adolescents in quality of friendships. On the other hand, a recent study comparing Dutch and North American 18–25-year old hearing and DHH college students who used a range of communication approaches (spoken or sign language or both) found that levels of quality of friendships and well-being in the two groups were similar (Blom et al., 2014). The lack of differences between DHH and hearing individuals on their quality of friendship contrasting the results of the other studies may be attributable to the participants being older and at a higher education level. In addition, unlike the three studies employing qualitative methodology or standardized assessments, Blom et al. (2014) used an online survey to explore quality of friendship.

Taken together, the few studies that compare the quality of friendship of DHH adolescents with that of their hearing peers suggest that DHH peers tend to report lower levels of positive features in their friendships but that friendship quality in DHH children should be considered in the context of their educational setting and their social-emotional skills.

## Factors Associated with Peer Relationships and Friendships of DHH Adolescents

### Gender

Studies exploring the relationships between gender and peer relationships of DHH adolescents are scarce and show mixed results. For example, a study exploring the effect of gender on peer acceptance and popularity of DHH adolescents using sociometric measures found that DHH girls were more popular and more accepted by their hearing peers compared to DHH boys (Wolters et al., 2012), whereas studies exploring relationships with hearing peers in mainstream educational settings (Martin & Bat-Chava, 2003; Wauters & Knoors, 2008) did not find an association between gender and peer relationships.

### Hearing Loss

It is expected that with the advances in technology (digital hearing aids and cochlear implants [CIs]) and early diagnosis, the degree of unaided hearing loss might not have an impact on peer relationships. Two studies (Antia et al., 2011; Marschark et al., 2007) explored the effect of degree of hearing loss on social skills and peer relationships of DHH adolescents and neither found an association between degree of hearing loss and peer relationships. It is argued that other factors (e.g., functional hearing, which is a measure of the students’ ability to use residual hearing with amplification within the classroom setting) might be better predictors of social-emotional outcomes than the degree of unaided hearing loss (Antia et al., 2011). However, a survey by Roberts and Rickards (1994) in Australia found that the degree of hearing loss was related to friendship patterns: the majority of children with mild to moderate hearing loss had mostly hearing friends, whereas those with severe to profound hearing loss had a more equal balance of hearing and DHH friends.

### Types of Amplification (Hearing Aids and Cochlear Implants)

There is growing evidence on the effect of CIs on the spoken language outcomes of DHH adolescents and on their social-emotional skills (Leigh et al., 2009; Moog et al., 2011). Studies in Australia (Leigh et al., 2009) and in Denmark (Percy-Smith et al., 2008) suggest that, compared to their hearing peers, DHH adolescents with CIs who communicated using spoken language and were in mainstream school settings manifested higher levels of social functioning than they did prior to the use of a CI, when they had been more likely to find communication with hearing peers challenging (Sahli & Belgin, 2006).

A recent study (Michael et al., 2018) using the parents’ version of the SDQ demonstrated better peer relationship outcomes for DHH children and adolescents with CIs compared to those who use hearing aids. Despite the recognized effects of cochlear implants on peer interaction, adolescents with CIs are a heterogeneous group demonstrating diverse experiences. Thus, Dammeyer, Chapman, and Marschark (2018) found that 55.4% of the participants felt different from others of their age, whereas 18.5% reported trying to hide their CIs often or all the time. However, adolescents in this study began using CIs later (mean age of CI surgery was 5 years) compared with today’s practice (below age of 12 months).

### Communication and Language Skills

Functional communication skills are dependent on language development and on experiencing models of good communication around the developing person (Wolters & Isarin, 2015). Compared to their hearing peers, DHH children who use either spoken or both spoken and sign language are less likely to be chosen by a hearing child as a friend (Nunes et al., 2001), tend to have no friends (Wauters & Knoors, 2008), and to be less popular (Peterson et al., 2016). According to an earlier study, adolescents who use only sign language in communicating with DHH and hearing peers who cannot sign, have a greater propensity to encounter peer problems when educated in a school that relies on oral communication in contrast to one that relies on non-oral communication (Vostanis et al., 1997). However, a recent study with DHH college students, some of whom were recipients of CIs, reported that social participation was not associated with the use of sign language or deaf acculturation nor with the use of spoken language (Marschark et al., 2018). These authors attributed this lack of association to the heterogeneity of the DHH population where there were multiple different factors underlying difficulties for individuals achieving social and academic success.

Furthermore, adolescents in mainstream schools with superior levels of spoken language and those in segregated educational settings with superior skills in sign language demonstrated fewer peer problems than their less skilled DHH peers (Fellinger et al., 2009). A longitudinal study of the same population-based sample from which participants in the present study were drawn reported concurrent correlations between spoken language and total emotional and behavioral difficulties on the SDQ scale, whereas superior spoken language or reading comprehension scores at 6–10 years was predictive of fewer emotional and behavioral difficulties at 13–20 years of age (Stevenson et al., 2018).

### Educational Setting and Hearing Status of Peers and Friends

One key factor in peer interaction and the preference of DHH adolescents for DHH or hearing friends is the school placement (Brice & Strauss, 2016; Israelite et al., 2002). Earlier and recent studies have demonstrated that DHH adolescents in schools for the deaf were more likely to have all or mostly DHH friends, whereas DHH adolescents in mainstream schools were more likely to have all or more hearing friends (Gregory et al., 1995; Millen et al., 2019). DHH adolescents in schools for the deaf show more difficulties in relationships with DHH and hearing peers than DHH children in mainstream schools or in specialist-support provisions (specialist units for DHH children attached to mainstream schools) (Musselman et al., 1996). Besides, a systematic review on the effect of education setting on social process and outcomes suggested that DHH children in mainstream settings perceive their relationships with hearing peers as less satisfying compared to their relationships with other DHH peers (Kluwin et al., 2002). It is important to recognize that since these studies were conducted, much has changed, but also some things have remained the same. Thus, when adolescents are in mainstream schools, they are often the only, or one of very few DHH people in the school. Their experience of interacting with hearing peers, although it may differ based on the support that their setting offers, is still different from those in schools for the deaf and specialist support provisions (Brice & Strauss, 2016).

## The Present Study

Our review of the literature indicated that DHH adolescents face more difficulties in peer relationships compared to their hearing peers and tend to report lower levels of positive features in their friendships. Using qualitative methodology, the present study explores how adolescents themselves view their relationships with peers and provides insight into the perceptions of peer problems as reported by DHH participants. DHH adolescents are a heterogeneous group. It is perhaps for this reason that previous studies revealed inconsistent results on the effect that individual characteristics (e.g., gender, degree of hearing loss, type of amplification, language and communication) and contextual factors (e.g., education placement) have on their peer relationships. To better understand the peer relationships and quality of friendship of DHH adolescents in relation to the adolescents’ individual characteristics and educational setting, the present study focused on the views and experiences of DHH adolescents themselves. This study aimed to develop a deeper understanding of the experience of their peer relationships and the quality of those relationships using semi-structured interviews in a representative sample of DHH adolescents. In addition, this study aimed to explore how the participants’ characteristics (e.g., level of hearing loss, emotional and behavioral health ratings, and language skills) contribute to their experience of peer relationships. Identification of the positive and negative experiences of peer relationships and friendships of DHH adolescents has the potential to inform the selection of those interventions more likely to be effective in supporting DHH adolescents’ social relationships and overall well-being. We elicited the DHH adolescents’ views to address three research questions:

1. How do adolescents who are DHH experience their relationships with peers and friends?
2. What are the positive and negative qualities of their friendships?
3. How do the DHH adolescents’ characteristics contribute to their experience of peer relationships?

## Method

### Participants

Participants of the current qualitative study were selected from a sample of DHH adolescents who had taken part in a wider quantitative study, which investigated language, reading, emotional, and behavioral well-being, and health economics in 76 DHH adolescents and 36 of their hearing peers drawn from a population-based birth cohort from Wessex and Greater London regions of the United Kingdom (Kennedy et al., 2006; Pimperton et al., 2016; Pimperton et al., 2017). Fifteen of the DHH group reported in that study, referred to hereafter as the 2017 study, had a long-term health condition additional to their hearing loss. In that study, the group mean score on the peer problems scale of the SDQ was higher in the DHH group than in the normally hearing group (Stevenson et al., 2017). Higher SDQ scores were associated with lower receptive language skills. For the present study, we invited all 61 DHH adolescents in the 2017 study who had no additional health condition to participate in this further study of their peer relations by taking part in semi-structured interviews. The findings reported here were from the 30 of these who accepted that invitation.

We classified participants as adolescents who use spoken language and those who use sign language. Six out of seven British Sign Language (BSL) users in the 2017 study participated in the present study. This coincided with our planned purposive selection of a sufficient number of signers to have a reliable indication of signers’ experiences of peer interaction, which we expected to be distinct from that of spoken language users because of their greater likelihood of self-identification with the Deaf world (Chapman & Dammeyer, 2017).

## Measures

### Qualitative Measures

*Semi-structured interview on peer relations and friendship*. Data were collected using semi-structured interviews that enabled participants to provide their own interpretation of peer interactions and to explore issues, which might not have been included in a predevised (structured) interview schedule (Cohen et al., 2013). The participants were thanked at the beginning of the interviews for agreeing to take part, were reminded about the topics of the interview, and were reassured that they could stop at any point or not answer any questions they did not feel like answering. At the end of the interview, they were given the opportunity to ask any questions or add anything that was not covered during the interview. Participants were given enough time, varying by participant, to answer the questions. This was approximately 5 min for each question. Questions unclear to the participants were rephrased.

This semi-structured interview schedule was adapted from existing inventories (Asher & Wheeler, 1985; Cassidy & Asher, 1992; Gregory et al., 1995; Skelton & Valentine, 2003; Wheeler et al., 2009) and included 21 questions on topics regarding experiences of DHH adolescents in school and relationships with peers and friends. The topics covered and indicative questions for each topic are presented in Table 1. Participants were then asked further questions to elaborate on specific issues.

**Table 1.** Topics of the semi-structured interview and indicative examples of questions asked

Topics of the interview	Examples of questions for each topic
Comparison between primary and secondary schools	Could you please compare your primary school experience with your secondary school?
Importance of school	What do you like and dislike about your school?
	How important is school for you? Why?
	Have you experienced any difficulties in school?
Relationships with peers	Are there any times that you went to your classmates to ask for advice or help on something?
	What do you think your classmates like about you?
Relationships with friends	What does being a friend mean to you?
	Are there any situations when it’s more difficult to make a friend than others?
	How important are friends for you? Why?
	What would you say that is special about [name of friend], that makes [name of friend] your best friend from school?
	What do you like doing with your friends?
Experiences of bullying	What does bullying mean for you?
	Has bullying ever happened in your school?
Being deaf	How do you see yourself regarding hearing loss?
	What would you like to tell people about a Deaf teenager’s life… so for example, if you could make a YouTube video… how would you make it, what would you include, what would you like to tell people about being a Deaf teenager?
	Do you think life would be different if you were hearing?

Before use with participants, the questions were piloted in a sample of six adolescents attending a school for the deaf using their preferred method of communication (spoken or sign language or both). Questions that were unclear to the pilot study participants were rephrased. Further questions were added based on themes that emerged during the pilot interviews.

### Quantitative Measures Used To Characterize Participants In This Qualitative Study

*SDQ*. The SDQ is a quantitative measure of emotional and behavioral health (Goodman, Meltzer & Bailey, 1998) that is widely used with children and young people. It is comprised of five scales (emotional symptoms, conduct problems, hyperactivity, peer problems, and a prosocial scale). Each scale has five items. The Total Difficulties score is derived by summing up the score of all the scales except the pro-social scale. For characterization of participants in the present study, scores were defined as normal or borderline or abnormal according to the three-band categorization suggested by the SDQ scoring guidelines.

*Hearing loss*. Severity of hearing loss was categorized as moderate (40–69 dB HL), severe (70–94 dB HL), or profound (≥95 dB HL) according to four-frequency averaging of the pure-tone thresholds at 0.5, 1, 2, and 4 kHz in the better ear.

*Receptive language*. Receptive vocabulary skills were quantified using a Receptive Language Composite standard score (RLC) calculated as the mean of standard scores on the Test of Reception of Grammar (TROG) (Bishop, 2003) and the British Picture Vocabulary Scale (BPVS) (Dunn & Dunn, 2009). These scores were available for all participating spoken language users but not for the six BSL users because these tests assess receptive skills of spoken English and not signing skills.

## Procedure

DHH adolescents with no additional medical conditions who had participated in the 2017 study were asked at that time if they were interested in being interviewed. The interviews were conducted either by the first author (an experienced teacher of the deaf) or by a research assistant (experienced in working with DHH individuals), both of whom were trained in qualitative methods. At each interview, there was always a participant and one interviewer, except with BSL users when a BSL interpreter was present as a third party. Written consent for participation was obtained from the adolescents and, if they were younger than 17 years of age, from their parent or guardian. The study was approved by the Southampton and South West Hampshire Research Ethics Committee. Adolescents were assured of the anonymity of the interview content and informed that they could stop their interview if they felt upset or if they did not wish to continue at any time.

Interviews were conducted using each adolescent’s preferred mode of communication (spoken English or BSL) and were audio-recorded (see below for procedure to achieve this for the BSL users). Although video recording was considered, it became clear from conversations held in the course of the 2017 study that potential participants were reluctant to be video-taped. We, therefore, deemed that video recording was not appropriate due to the personal and sensitive nature of the conversations.

For the BSL users, a communication protocol was based on the following practices as described by Davis (2005): (a) everything that was signed was interpreted (including side comments); (b) a highly qualified (Signature Level 6 NVQ Certificate) registered BSL interpreter, with whom the communication protocol had been discussed to ensure accuracy of interpretation, was present to provide a voice-over onto tape; (c) the interpreter sat opposite the DHH participant and next to the hearing investigator so that eye contact could be maintained and there was enough light to watch the interpreter’s face; (d) facial expressions were used to convey both emotional affect and grammatical content; and (e) sufficient time was allowed at the beginning of the interview to enable the DHH adolescent and the researchers to become familiar with each other’s communication style. Although it can be argued that the presence of a third party (interpreter) might have hindered the communication dynamics between the interviewer and the interviewee, the use of an experienced interpreter made the content of the interviews accessible. In addition, participants, whose preferred mode of commutation was BSL, informed the researchers prior to the interviews that the use of an interpreter was necessary for their communication needs to be met.

## Data Extraction Process from Interviews

All interviews were transcribed by the first author and by a professional experienced in speech transcription of DHH adults (i.e., a transcription service was used and paid for). Transcribed interviews were entered into NVivo10 (https://www.qsrinternational.com/nvivo/what-is-nvivo) to assist with data handling, organization, and coding. Thematic analysis was used to identify, analyze, and report themes within data following six steps: (a) familiarization with the data, (b) generation of initial codes, (c) search for themes, (d) review of themes, (e) defining and naming themes, and (f) report writing (Braun & Clarke, 2006). Identification of themes for thematic analysis was both inductive (themes strongly linked to the data themselves) and theoretical (themes driven by the researchers theoretical interest) (Braun & Clarke, 2006). Themes relating to peer problems were thus identified theoretically, based upon the SDQ peer problems scale (e.g., being picked upon or bullied, feeling lonely, being liked by peers, having friends) and themes relating to positive and negative aspects of friendship based upon the friendship quality features identified by Berndt (2002). Themes relating to DHH adolescents’ relationships with their peers that did not fit in these theoretically derived themes also emerged inductively from the data. Themes were developed and revised in an iterative manner as patterns within the data became more apparent. Investigators met regularly to discuss patterns and emerging themes in the data and to reach consensus on the major themes.

The first author undertook initial coding (i.e., identification of themes) of the interviews. Two independent raters not involved in the data collection, a psychologist ([JK]), and a speech and language therapist (SW), both experienced with the deaf community as researchers and practitioners, re-coded all (SW) or 20% (JK) of the interviews. Inter-rater reliability was calculated in NVivo10 for each piece of coding (themes) and for each transcript. Inter-rater agreement was assessed in NVivo10 by calculating the percentage agreement (i.e., the number of units of agreement divided by the total units of measure within the data item, expressed as a percentage) between raters for each individual combination of coding and transcript, as recommended by Noble and Smith (2015). Agreement for the major themes ranged from 97% to 99%.

## Results

Participants ranged in age from 13.7 years to 19.3 years with a mean (SD) age of 16.6 (1.37) years with equal numbers of males and females (Table 2). Six out of 7 (86%) of participants who used sign language and 24 out of 54 (44%) of participants who used spoken language in the 2017 study participated in the present study. Because of this purposive over-representation of signers, participants in the present study included a higher percentage with severe/profound, rather than moderate, hearing loss (67 vs. 33%, χ^2^ [2, *N* = 61] = 6.5, *P* = 0.03) than was the case among 2017 study participants that were eligible but did not participate in the present study. There were, however, similar percentages of participants and eligible non-participants with severe/profound hearing loss who used spoken language. Twenty-one (70%) participants wore digital hearing aids and nine (30%) were fitted with unilateral or bilateral CIs (Table 2).

**Table 2.** Characteristics of DHH participants: gender, severity of hearing loss, educational setting, mode of communication, type of amplification

Characteristic		DHH participants (%) *N* = 30
Gender	Male	15 (50%)
	Female	15 (50%)
Severity of Hearing loss	Moderate	10 (33%)
	Severe/profound	20 (67%)
Educational setting	Mainstream	18 (60%)
	Specialist support provision	4 (13%)
	Schools for the DHH	8 (27%)
Mode of communication	Spoken English	24 (80%)
	BSL	6 (20%)
Type of amplification	Hearing aids	21 (70%)
	Cochlear implants	9 (30%)

Participants ranged in age from 13.7 to 19.3 years with a mean (SD) age of 16.6 (1.37) years with equal numbers of males and females.

DHH = Deaf or hard of hearing; BSL = British Sign Language.

Specialist support provision = specialist units for deaf and hard-of-hearing children attached to mainstream schools.

Eighteen (60%) participants were educated in mainstream settings (Table 2), 8 (27%) in schools for the deaf, and 4 (13%) in specialist support provisions (specialist units for DHH children attached to mainstream schools). The present sample did not differ significantly with respect to their educational settings from the 31 participants in the 2017 study that were eligible but not included in the present study (χ^2^ [2, *N* = 61] = 0.91, ns).

The DHH spoken language users (*N* = 24) in the present study did not differ significantly from the spoken language users (*N* = 30) who did not participate with respect to their self-ratings on SDQ scales or subscales (χ^2^ [1, *N* = 54] = 0.80 to 1.63, ns in all cases) (Table 2). Compared to the percentage of SDQ scores that were borderline/abnormal in the reference community sample on which the SDQ was validated (Goodman, Meltzer & Bailey, 1998), the percentage of borderline/abnormal scores in the present study was higher on the Peer Problems subscale (30 vs. 20%) but similar on the other subscales (13–20 vs. 20%). The mean (SD) Receptive Language Composite standard score (see Methods section) of the users of spoken language in the present study was similar to that of the eligible non-participants in the 2017 study (*t* (54) = 1.13, ns) with a mean score of 90.5 (i.e., in the low average range) a standard deviation of 12.54 and range of 62 to 106 (Table 3).

**Table 3.** Distribution of normal and borderline/abnormal strengths and difficulties Questionnaire scores in DHH participants and non-participants^*^ in the present study

	All participants		Oral language users only			
	*N* = 30		Participants (*N* = 24)		Non-participants (*N* = 30)	
SDQ scales	Normal *N*	Brdrline/Abnml *N* (%)	Normal *N*	Brdrline/Abnml *N* (%)	Normal *N*	Brdrline/Abnml *N* (%)
Emotional symptoms	26	4 (13)	23	1 (4)	28	2 (7)
Conduct problems	24	6 (20)	21	3 (13)	29	1 (3)
Hyperactivity	26	4 (13)	23	1 (4)	25	5 (17)
Peer problems	21	9 (30)	19	5 (21)	25	5 (17)
Total difficulties	24	6 (20)	21	3 (4)	28	2 (7)
Prosocial	25	5 (17)	22	2 (8)	28	2 (7)

DHH = Deaf or hard of hearing; SDQ = Strengths and Difficulties questionnaire (Goodman, Meltzer & Bailey, 1998).

^*^i.e., DHH participants in the Stevenson et al., 2017 study with no health condition additional to their hearing loss that did not participate in the present study.

## Interviews

The qualitative analysis of peer problems resulted in three major themes: experiences of relationships with peers, positive aspects of friendship, and negative aspects of friendship. At least one of the three identified themes emerged from the interview transcripts of all participants. There were 11 subthemes within these three themes (Table 4). Table 4 presents the numbers of participants in the present study in whom the individual subthemes emerged. The findings for each theme are presented using illustrative quotations (the exact words of the adolescents) alongside the participant’s sex, age, mode of communication (spoken language or BSL), educational setting, and degree of hearing loss (HL).

**Table 4.** Numbers and percentages of participants who reported experiences in each theme and subtheme

Major themes	Subthemes	*N* (%)
Experiences of relationships with peers	Feeling accepted	21 (70%)
	Ease of making new friends	22 (73%)
	Barriers in making new friends	13 (43%)
	DHH friends, hearing friends, or no preference	5 (16%)
	Feeling different from peers	19 (67%)
	Experience of being bullied	23 (77%)
Positive aspects of friendship	Similarities or common interests	21 (70%)
	Intimacy	22 (73%)
Negative aspects of friendship	Conflicts	15 (47%)
	Lack of interaction	18 (57%)

### Experiences of relationships with peers

Experiences of relationships with peers emerged from the interview transcripts of all participants and could be divided into six subthemes: (a) the experience of feeling accepted, (b) ease of making new friends, (c) barriers in making new friends, (d) preference of friends based on their hearing status, (e) the experience of being the only DHH person in a mainstream class, and (f) the experience of being bullied.

*Feeling accepted*. Feeling accepted was defined as feeling part of the class, being at the same achievement level as their peers and being able to ask for help when needed:

“*Yes, most of my friends, like we were roughly around the same level so we were mostly the same classes. And yeah, everyone just helps each other and stuff. Teachers are also supportive and helpful” (*Female, 16 years, spoken language, mainstream, moderate HL).

Feeling accepted in class was not only attributed to the good relationships with peers but also to the supported environment provided by teachers and support staff.

Good relationships with peers were reported by 21 of 30 (70%) participants, they described their classmates as nice, feeling able to ask them for help with work when needed or to seek clarification from them when they had not heard the teacher’s instructions.

“*Obviously, if I didn’t hear anything that the teachers told us to do, what we need to do, then I will ask my friends to explain what we have to do, that’s one thing. The second thing is making sure that I’m writing the right thing, rather than like knowing what to do, but I’m writing completely off the topic.”* (Male, 18 years old, spoken language, mainstream, severe HL).

In response to the question: “What do you think your classmates like about you”? participants commented that they thought they were liked by peers because of their good behavior in the classroom, good academic achievement, and positive traits of their character (e.g., being “f*unny,*” “*trustworthy,*” “*smiley*” or “*polite*”). These statements were provided by 23 (77%) participants who explained that they were liked by their classmates because they possess a number of positive character traits:

“*I’m pretty funny, and I think one of the most important things, I’m trustworthy, because I do have quite a few friends from here who tell me everything, cos she has an interesting life compared to me, but she tells me everything that’s going on with her life, and I’m like, “Oh my God,” and I give her advice, but I don’t tell anyone because she only tells me*.” (Female, 17 years old, spoken language, mainstream, profound HL).

*Ease in making new friends*. Twenty-two of the 30 (73%) participants reported no difficulty in making friends. The ease of making friends depended on the situation and the environment in which this was taking place, with school being the easiest setting.

*“At school, I wouldn’t say it is difficult because if you are with your mates and you make a mistake they can back you up. Like I am very confident when it comes [to] the group of people in school and talking to people from school.”* (Female, 16 years, spoken language, specialist support provision, severe HL).

In the school environment, adolescents felt confident in talking to their peers and expanding their circle of friends. Participants reported that it was easier to make friends in their school environment compared to other social situations (e.g., during school holidays). They also shared their anxiety about making friends beyond compulsory education, and when entering further education. However, those participants stated that it was easier to make new friends and to connect with their friends on social media:

*“I have like 250 friends on Facebook. I just add them on Facebook and just start talking. I think you only have to do is ask someone on Facebook and then you* can *find out everything there is to know about them, you know, you can go through all their photos and say oh, this person’s like that and you can sort of go on that”* (Male, 17 years, spoken language, mainstream, moderate HL).

Connecting with people and making friends through social media seemed to be easier for adolescents who felt less confident in social situations outside their school setting.

*Barriers in making new friends*. Thirteen out of 30 adolescents (43%) found it hard to make new friends and did not feel confident to speak to new people. Eight of those 13 adolescents had moderate hearing loss, three had severe and two profound hearing loss. Difficulty being understood by others resulting in low confidence was a common reason given by the adolescents to explain their difficulty making new friends.

“I do find it difficult to make friends because I’m quite a very shy person and I don’t have that much confidence in speaking up to new people. Sometimes when I meet new friends, sometimes they do not understand me because, obviously, when I was born, I was not meant to have my voice, but I’ve like have a speech therapy to improve my voice, talking (Male, 16 years, spoken language, mainstream, severe HL).”

Besides communication issues, disclosure of the adolescent’s hearing loss was identified as an additional barrier to establishing new friendships. Some adolescents who disliked being labeled “Deaf” disliked disclosing their hearing loss.

“*Yeah, so I did find it a bit scary making friends. But when I meet new... Even now, sometimes when I meet a new group of people, like a mixture of guys and girls, I can be a bit shy because none of them know that I have a hearing loss. It can be a bit... It’s hard work sometimes for me, but it’s fine ….I consider myself as hearing impaired I prefer that term because deaf is like fully. All my friends consider me as hearing”* (Male, 17 years, spoken language, mainstream, moderate HL).

In addition, a link was made between not wishing to be identified as Deaf, and hearing loss being perceived as an initial barrier to making friends. Adolescents who experienced problems making new friends because they did not wish to disclose their hearing loss and did not want people to take notice of their hearing loss. Those adolescents identified themselves with the hearing world.

*DHH friends, hearing friends, or no preference*. Participants, irrespective of their educational placement or severity of hearing loss, reported having both DHH and hearing friends. The communication mode and the world with which they identified (Deaf or hearing) were the main reasons given for their preference for hearing or DHH friends. Adolescents who communicated using spoken language tended to prefer to have both DHH and hearing friends but explained that it would be difficult to be friends with Deaf people whose main method of communication is signing.

*“It depends where you are to be honest, if you’re in the Deaf world it’s like harder for me ‘cos I can’t really sign that well.*” (Male, 17 years, spoken language, specialist support provision, moderate HL).

For some adolescents having hearing friends was a way to be included and accepted in the hearing world. Mixing with hearing people was also considered important for later life:

*“I tend to hang out with hearing people because there is more a range of hearing people, more mixed, and you have to get used to it because if you go to work you have to be with hearing people so you have to get used to talk to them.”* (Female, 16 years, spoken language, specialist support provision, profound HL).

Three signers had only Deaf friends. Those adolescents embraced their Deaf identity, were involved in Deaf clubs and Deaf culture, and felt that they could only feel connected to other DHH people. Deaf friends were perceived by these participants as sharing a similar self-image; sharing the same language and the experience of being DHH were seen as attributes conducive to friendship:

*“So the person that is not deaf and is hearing, I find that very very difficult to make friends with that person…. With deaf people is easier because I already share something….the same language. I think I know that a deaf person will understand me”* (Male, 17 years, BSL, school for the deaf, profound HL).

In summary, communication and a wish to be connected to either the hearing or the Deaf world or both were the adolescents’ stated reasons for having DHH or hearing friends.

*Feeling different from peers*. The only situation when adolescents reported feeling excluded from their class and unsupported by their peers was when the student was the only DHH student in the classroom (at a mainstream school). This experience was reported by 5 of 18 (27%) adolescents with a moderate hearing loss who were in a mainstream classroom with no DHH peers.

*“At times I do wish there was like another person who is hearing impaired at [name of the school] like there isn’t one at the moment. I think I’m like my school’s first hearing impaired so it’s quite new for them.”* (Female, 15 years, spoken language, mainstream, moderate HL).

The aesthetic appearance of their hearing aid and having to ask teachers to use an FM system (the wireless assistive hearing device that enhances the sound of hearing aids or CIs) were the main reasons most often given by participants for feeling different from their hearing peers.

“*I don’t... I actually do not know what it was, it might have been that but I just hated it (i.e., FM system), I just didn’t like... [pause]I think it’s probably just being different and like having to wear it, you know.”* (Female, 16 years old, spoken, mainstream, moderate HL).

*Experience of being bullied*. In response to the question “What does bullying mean for you”? the adolescents described bullying as behaviors that can hurt other people either physically or emotionally. Adolescents considered a “nasty” behavior as bullying even if it only happened once. However, a further distinction was made between bullying and teasing. Teasing was described as “making fun” of or “playing pranks” on other people.

The adolescents described situations of being teased in secondary school but reported this behavior as acceptable:

*“I have been teased but not bullied. . . I wouldn’t call it bullying. Just tease names like “Can you hear”? My friends say a lot but I can deal with that because I know they are joking.”* (Male, 15 years, spoken, mainstream, severe HL).

One or more incidents of being bullied in primary school were reported by 23 of 30 (77%). All participants reported that bullying had now (in secondary school) stopped. Thus, all the quotes presented below relate to experiences of being bullied in primary school. Among spoken language users, the group mean Receptive Language Composite standard scores of participants that reported that they had been bullied in primary school did not differ from that of those who did not report this (*t*(24) = 0.69, ns).

Reasons for being bullied varied: some adolescents were not always able to identify the exact reason for being bullied but others attributed it to their hearing loss, communication difficulties, or reasons unrelated to being DHH, such as having a different religion:

“*Because my best friend is same like me, she’s deaf, but she’s very good hearing, she always told me that all hearing people been talking behind my back about me all the time. ….I was not sure if I should listen to her …… [but] one day when I went to my classroom all hearing [people] teasing my [deaf] group punched me. Then I realised she was right*.” (Female, 15.3 years, spoken language, school for the deaf, profound HL).

Being bullied had an effect on DHH adolescents’ self-esteem making some of them feel depressed and isolated, whereas others felt angry:

“*It can just, I don’t know, it makes me feel angry. Sometimes it makes me feel, it makes me feel, you know, a bit isolated from them because, obviously, they don’t, they haven’t got any disability or any impairments or anything, so they just, you know, they’re alright, they’re perfectly normal.”(*Male, 15 years, spoken language, mainstream, moderate HL).

The majority of young people described verbal bullying, including gestures, but physical bullying was also reported:

*“They called me names.. they grabbed my chair.. they put a broken pen on the chair… they take my.. books so I cannot find them.. and they got me into trouble”* (Female, 17 years, spoken language, specialist support provision, severe HL).

Adolescents had been bullied by hearing peers but one participant also described her experience of being bullied by DHH peers.

“*There was a Hearing Impaired Unit there, and the numbers fluctuated over the years so I couldn’t tell you how many people were there…..But I was definitely bullied there, it wasn’t just the hearing children that bullied me, the deaf bullied me as well, and the deaf were the worst culprits…..I thought they were my friends at the time and that did upset me, it made me, it made me feel that I was unimportant at the time.”(*Female, 19 years, BSL, Mainstream, profound HL).

All six adolescents who used BSL as their preferred method of communication reported that they had been bullied in primary school. Those who received their primary school education in mainstream schools attributed the cessation of bullying to their move to a school for the deaf.

“Not really, [it was in primary school] and I couldn’t, you know, verbalise back, I just used to sit, I was one Deaf person, they were all hearing there and there was a lot of teasing of me and I couldn’t go back there and I just used to sit there and take it and hence my confidence is very low”. (Male, 16 years, BSL, school for the deaf, severe HL).

### Positive qualities of friendship

Twenty-two (73%) participants reported experiencing positive qualities of friendships (similarities or common interests, feelings of intimacy).

*Similarities or common interests*. Participants described feeling close to friends with whom they shared hobbies, were involved in the same activities, and shared love for the same things in life. For example, one participant described how love of music brought her and her friend close to each other.

*I mean (friends’ name) and I have a big thing in common which is music, you know we really like music, he’s really good at playing, I am not so good at playing but we both love, you know, sort of music*. (Male, 16 years, spoken language, school for the deaf, profound HL).

Attending deaf clubs and having an interest in the life and activities of the Deaf community were particularly important for maintaining a close relationship for deaf adolescents who signed and identified themselves as belonging to the Deaf world:

*“We actually like going to the (name) Deaf Community because like when we were in like Year 6 we used to go on trips, like all deaf children.”* (Male, 16 years, BSL, school for the deaf, profound HL).

#### Intimacy

Another positive quality of friendship was the feeling of intimacy. This was characterized as experience of situations in which friends shared personal information, feelings, and secrets. Discussions about family issues were indications of feelings of intimacy between adolescents and their friends.

*“And her Mum and Dad are divorced so sometimes, even though they still get on, there’s sometimes, you know, like sometimes she’ll be a bit like, oh my Dad’s, you know...So when this happened I comforted her and when my dog died, she obviously comforted me. So, you know.”* (Female, 15 years, spoken language, mainstream, moderate HL).

Discussion about difficulties that participants and their friends had experienced within intimate relationships and disclosure of conflicts that they had with other people were also indications of the intimacy that they felt with their friends.

*And she had a problem with her boyfriend, so I’m like... And we’re like, “OK, fine, don’t worry about this guy. He’s some idiot, he’s some something,” you know... there’s more fish in the sea.”* (Female, 16 years, spoken language, mainstream, profound HL).

### Negative qualities of friendship

The majority of DHH adolescents (72%) also reported some negative experiences in their friendships. These negative experiences were classified as conflict and as losing contact with friends.

*Experience of conflict*. The DHH adolescents described feelings of anger leading to potential conflicts with their friends. Conflicts were attributed to a variety of causes, such as negative characteristics of a friend, inability to keep secrets, and betrayal of trust. This included situations when their friends thought that they had betrayed their trust:

*Mainly about 3 years ago, me and my friend who were sharing the room we had a conflict. . . We were always quite close but you know sometimes people hear a rumour and then you don’t really understand what happened and then it became awkward. . . I don’t really know what happened but she talked about her family so I haven’t said anything about her family but that is what she thought. . . Deaf people like talk about others the whole time*. (Female, 17 years, spoken language, school for the deaf, profound HL).

Participants often described their relationships as good but sometimes damaged by a single event that was perceived as a sign of betrayal of friendship. However, in most situations, the adolescents described betrayal of trust as being a temporary conflict:

*“It is probably... that was before the Easter holidays and me and some of my friends were all going to…. and shared a room pretending that nothing had happened and pretending that everything is fine.. we can do that.. we can retain the friendship.. It wasn’t a horrible experience.. but I think she should say sorry but she didn’t. I think she has to say it because* …”(Female, 16 years, spoken language, mainstream, moderate HL).

Gender differences were observed with 10 of 15 (67%) females describing one or more incidents of arguments/conflicts as a feature of their long-lasting friendships compared with 5 of 15 (33%) boys.

*Lack of interaction*. Participants described situations when they drifted apart from their friends, not because of conflicts but due to lack of interaction. Lack of interaction was attributed by the adolescents to their friends having fewer opportunities to interact with them. They reported lack of interaction in cases where their friends lived far away or when they attended different classes in school. In instances when their friends had moved schools or moved house (to a more distant place), lack of time to engage with their friends outside school had led to a decrease in opportunities for interaction.

The main reason for fewer opportunities for interaction reported by 10 out of the 18 DHH adolescents was the situation in which their friends had built a relationship with another peer. Adolescents described feeling rejected, hurt, and lonely in these instances:

*Me and [name of friend] are... we didn’t talk... We were really close and we’re drifting apart because she’s got close to another girl, and I was thinking, “Oh God, I’ve got... I miss her.” And we don’t talk when we see each other, we never talked that much, so she’s like... she’s like talking to me as if I was kind of a new girl or something, and like, “Mate...” you know.* (Female, 16 years, spoken language, mainstream, profound HL).

*And well I think he didn’t want to be friends with me one day, I didn’t know why and then...He went down to college and standing near a group of friends, so I was a little bit surprised and thought what’s going on? I think now he wants to be friends again but... if he starts talking to me I will talk back but I wouldn’t be friends with him again. I don’t trust him with my personal stuff but…* (Male, 17 years, spoken language and BSL, school for the deaf, profound HL).

However, none of the 10 adolescents (56%) who reported that the lack of interaction was due to their friend forming a new relationship gave any indication on whether the new relationship that their friends developed was with a DHH or a hearing peer.

## Discussion

This is, to our knowledge, the first qualitative exploration of relationships and friendships in a sample of DHH adolescents drawn from a population-based cohort study (in the United Kingdom) in which their receptive language and social–emotional skills had been assessed. This is the first and only report of the qualitative findings. The main findings and contribution of this study to existing knowledge are threefold. Firstly, overall DHH adolescents experience positive relationships with their peers and friends notwithstanding their earlier experience of bullying in primary school. Secondly, the majority of adolescents with moderate hearing loss experienced barriers in making friends, felt different from peers in a mainstream classroom due to the physical appearance of hearing technologies and found it easier to make friends in school settings and through social media than in other social situations. Thirdly, DHH girls were generally found to experience more conflicts in their friendships than boys suggesting that girls might benefit from targeted support on developing their peer relationships.

### Peer relationships

In contrast to a previous study in which adolescents reported feeling worried about peer relationships and struggling with their concept of self (Punch & Hyde, 2011), participants in our study report that they felt accepted by their peers, experienced mainly positive relationships and felt able to ask for help from their friends. They identified the supportive environment provided by the teaching staff and the positive traits of character of the DHH adolescents as factors contributing to this.

Participants’ reports of being rejected by peers and being bullied in primary school are consistent with the report by Nunes and colleagues that DHH children feel neglected in inclusive settings (Nunes et al., 2001). The cessation of bullying in secondary school is consistent with other descriptions of DHH adolescents reporting improvements in their social experiences and better coping strategies with increasing age (Antia et al., 2011; Batten et al., 2014). With increasing age, participants in the present study were able to make a distinction between bullying and teasing, develop better coping strategies, and recognize which behaviors were acceptable and which were not.

### Quality of friendship

Almost three quarters of participants identified similarities or common interests and intimacy as positive qualities, and conflicts and lack of interaction with their friends as negative qualities of their friendships. The finding that a higher percentage of girls than boys experienced conflicts with friends, arising from issues of mistrust and infrequency of interaction, is likely to reflect the importance that girls place on close and exclusive social relationships and their greater dependence on peer feedback to inform their self-worth (Casey-Cannon et al., 2001).

Common interests, hobbies, and identification with the Deaf world as well as sharing of personal thoughts and feelings related to family and intimate relationships were considered to reflect positive qualities whereas betrayal of trust and lack of interaction with friends due to the formation of a new friendship reflected negative qualities of friendships. These more specific and detailed descriptions of positive and negative experiences with peers and friends have not been identified by previous quantitative studies and may help in the design and provision of effective and timely support.

### Factors affecting peer relationships and friendships

Feelings of being different from peers and excluded from their mainstream class (because of the assistive audiological equipment) were reported by adolescents with moderate hearing loss. Difficulties in being understood by their peers confirm earlier reports of the impact of atypical speech and expressive language used in conversation with peers (Fellinger et al., 2009). This is also consistent with findings from studies on younger DHH children with moderate hearing loss that report their risk of social-emotional difficulties as no lower than that of their peers with severe or profound hearing loss (Laugen et al., 2016). Most adolescents with moderate hearing loss in the present study reported greater ease in making new friends in their school setting than in other social settings because their hearing loss is already known. In addition, they were particularly comfortable on social media because their hearing loss is invisible and not identified at all in this setting.

The type of communication preferred by DHH adolescents (speech or sign) was identified as a factor affecting the choice of friends of DHH adolescents. Previous studies have also reported that communication type is a strong predictor of choice of DHH or hearing peers as friends (Antia et al., 2011; Maxwell-McCaw & Zea, 2011; Nikolaraizi & Hadjikakou, 2006; Wauters & Knoors, 2008). Deaf culture and Deaf identity were experienced as positive features in the friendships of BSL users, which were exclusively with DHH peers. Adolescents who used spoken language preferred to identify with hearing rather than DHH peers. For those adolescents, early identification, improved hearing aid technology, including CIs, and support for speech development may have contributed to their preference.

The lack of a relationship between the receptive language skills of DHH adolescents and their experience of bullying contradicts earlier findings suggesting that poor receptive language ability accounts for higher levels of emotional and behavioral difficulties (Fellinger et al., 2009; Stevenson et al., 2017). A possible explanation provided by Batten et al. (2014) is that other factors, such as ability to improvise in conversations and pragmatic language skills (not measured in the present study) could contribute to positive peer relationships.

### Strengths, limitations, and recommendations for future research

A strength of the study is the fact that those studied are representative of the birth cohort from which they were drawn and this is supported by the similarity of their educational settings to the distribution of educational settings reported for DHH adolescents in England by the Consortium for Research on Deaf Education (NDCS, 2017).

The verbatim descriptions and qualitative analysis reported here (e.g., experience of bullying, conflicts, and infrequency of interaction with their peers) complement our report of the reading and language skills (Pimperton et al., 2016, 2017) and propensity to peer relationship problems (Stevenson et al., 2017, 2018) of the study participants and of the hearing comparison group reported in the related quantitative study. Thus, the current study is one of the very few studies providing in-depth qualitative explanation of the data obtained by standardized assessments.

Bias regarding the interpretation of adolescents’ experiences was minimized by using highly qualified interpreters and independent raters to re-code transcripts. The purposive over-representation of BSL users is also a strength of the study, allowing us to capture the range of experience of both this distinct group and also of the spoken language users who constitute the large majority of all DHH adolescents.

The absence of a comparison group of hearing adolescents was a limitation because it would have enabled us to determine whether the percentage of adolescents who reported feeling accepted by their peers differed between hearing and DHH adolescents or whether experiences reported in the present study, such as greater prevalence of peer conflicts among girls than boys, are unique to DHH adolescents. We do, however, know from the related quantitative study that the group of DHH adolescents, of whom participants in the present study were a representative sample, had an elevated rate of borderline/abnormal scores on the peer problems subscale of the SDQ whereas the hearing comparison group did not (Stevenson et al., 2017).

The absence of video recording of the interviews is also a limitation but was the only course consistent with the expressed views of the participating adolescents who may, as a result, be a larger, more representative sample who were more willing to talk candidly on sensitive topics.

An additional limitation is that no connection was made between the adolescents’ experiences and family variables that might have influenced those experiences. Family characteristics with regard to hearing status (e.g., hearing, DHH), type of communication (e.g., ability to sign), and communication quality were recorded, but numbers in most of these subcategories were too small to allow examination of the associations between these variables and experiences with peers. Future studies should address family variables in relation to DHH adolescents’ experiences of relationships with peers.

### Implications for educational practice

The findings of the present study show that difficulties with peers and friends are important for DHH adolescents and might suggest that proactive promotion of communication and positive peer relationships between DHH and hearing adolescents can be potentially beneficial. The negative long-term legacy of bullying on the self-esteem of DHH adolescents (Hadjikakou & Papas, 2012) also suggests the need for more deaf awareness training in educational settings to improve confidence and skills of hearing individuals to communicate with their DHH peers. Deaf awareness training, however brief, would especially benefit the hearing students in mainstream classrooms (where there are only one or two DHH children) making them aware of the communication and social emotional needs of DHH children (Hindley, 2005).

A clear implication of the present study is that schools and teachers should pay more attention to the subgroup of adolescents who have moderate hearing loss. As found in the current study, this subgroup experience the same levels of difficulties as those with severe or profound hearing loss, but the former is often a “neglected” group as the perception is that they “can hear” (Antia et al., 2009). Thus, it is important that hearing peers, and trainee and newly qualified teachers receive guidance and training on how to interact with adolescents with moderate hearing loss and how to better support them in mainstream settings. Training of hearing peers and teachers is important in order to create a more inclusive social environment for those who have hearing loss. Specialist teachers (qualified teachers of the Deaf) have the knowledge and the responsibility to educate mainstream teachers regarding the needs of children and adolescents with moderate hearing loss in mainstream classrooms.

Peripatetic teachers of the Deaf (teachers of the Deaf visiting DHH children and adolescents in mainstream schools) can support development of effective school strategies for social inclusion and social functioning of DHH children and they too would benefit from further training to consolidate that supporting role. In mainstream schools, support for DHH children’s social-emotional development can come from a variety of school staff (e.g., the family liaison officers, teaching assistants, speech and language therapists, and Special Educational Needs Coordinators) other than just the teachers of the Deaf or the mainstream teachers, all of whom would benefit from training in deaf awareness and communication tactics.

## Funding

This work was supported by the Wellcome Trust (089251).

## Conflict of interest

No conflict of interests were reported.
